# Analysis on the Seismic Performance of Steel Fiber-Reinforced High-Strength Concrete Beam–Column Joints

**DOI:** 10.3390/ma14144016

**Published:** 2021-07-18

**Authors:** Ke Shi, Mengyue Zhang, Tao Zhang, Ru Xue, Pengfei Li

**Affiliations:** 1 School of Civil Engineering and Architecture, Zhengzhou University of Aeronautics, Zhengzhou 450046, China; shike@zua.edu.cn (K.S.); zhangmengyue@zua.edu.cn (M.Z.); xueru6239@163.com (R.X.); lipf2021@163.com (P.L.); 2 State Key Laboratory of Green Building in Western China, Xi’an 710043, China

**Keywords:** beam–column joints, steel fiber-reinforced high-strength concrete, finite element model, cyclic loading

## Abstract

The present research study aims to investigate numerically the behavior of steel fiber-reinforced high-strength concrete (SFRHC) beam–column joints (BCJs) under seismic action. Based on the plastic damage constitutive model of concrete and elastic–plastic mixed-strengthen constitutive model of steel material, the finite element software ABAQUS was utilized to establish the 3D finite element (FE) model of BCJs. Additionally, the feasibility and accuracy of the numerical simulation were verified by comparing the computed results and experimental observations in terms of the hysteresis curves, skeleton curves, and failure mode. Furthermore, based on the validated FE modeling approach, load vs. displacement hysteresis curves of SFRHC–BCJs during the loading process were analyzed in detail; the failure process was also investigated. Furthermore, the effect of various parameters on the seismic behavior of BCJs was analyzed comprehensively, including the concrete strength, the volume ratio of steel fiber, and the stirrup ratio in the core area. Finally, parametric studies illustrated that increasing the concrete strength helps in enhancing the ultimate load, while the ductility decreased noticeably. Both adding the steel fiber and increasing the stirrup ratio can significantly improve the seismic performance of BCJs.

## 1. Introduction

Beam–column joints (BCJs) are the critical parts of the frame structure, and their failure can easily lead to the collapse of the whole structure. Previous tests [1,2,3,4,5,6] showed that cracks were easy to occur in the core area of joints under earthquake load. To prevent brittle failure and improve ductility and bearing capacity, the current code of practice [7,8] stipulates the dense arrangement of shear connections. However, this may lead to blockage of steel bars, resulting in difficulties in the concrete pouring and increasing construction costs [9,10,11]. This is an excellent method to add randomly distributed short fibers into the concrete matrix. Discontinuous discrete fibers are completely integrated into concrete mixture in random directions, which has significant advantages for the performance of reinforced concrete members. Issa et al. [12] and Zhu et al. [13] obtained a higher ultimate compressive strain of concrete by adding steel fiber, thus improving ductility and flexural strength of fiber reinforced concrete beams. Hammad [14] studied the properties of structural polypropylene and steel fiber reinforced alkali slag concrete cured at room temperature. Horňáková [15] studied the effect of steel fiber on concrete electrical resistivity. Henager [16] found that adding steel fiber improved the corner ductility coefficient of BCJs, the bond strength of longitudinal reinforcement, and the ultimate bending moment. Filiatrault et al. [17] conducted experiments on steel fiber reinforced concrete side column joints under low cyclic loading. Compared with nonfiber concrete BCJs, the results show that high steel fiber content concrete BCJs have a higher ductility coefficient and stronger energy dissipation capacity. Shannag [18] found that compared with reinforced concrete BCJs, the energy dissipation capacity of SFRHC beam–column joints increased by 20 times, and the stiffness degradation decreased by two times. Ganesan [19] analyzed the influence of steel fiber volume ratio change on the seismic performance of BCJs through 10 reinforced steel fiber-reinforced high-strength concrete BCJs tests. It is found that ductility, energy consumption, and shear capacity of BCJs increased with the increase in steel fiber volume ratio. Li [20] tested a series of fiber-reinforced high-strength concrete beams under static and explosive loads using impact tubes. The results show that fiber can significantly improve the ductility of high-strength reinforced concrete beams under static load. Under dynamic conditions, the application of fiber-reinforced high-strength concrete can significantly improve the blasting performance. Gencoplu [21] et al. designed seven full-scale BCJs and studied the influence of stirrup ratio and steel fiber addition range at the beam end on the seismic performance of the specimens by pseudo-static loading to determine the best steel fiber addition range at the beam end.

Over the past years, experimental studies have always been a basic analysis method for investigating the seismic behavior of RC structures. However, with the advancement of computing techniques to conduct research more efficiently and economically, numerical analysis is becoming more and more popular to simulate the behavior and predict RC structures’ response because it can simulate the case that is difficult and/or complex [22]. Bui et al. [23] studied the BCJs under the unloading–reloading cycle experimentally and numerically. Abbas [11] used the finite element software ABAQUS to carry out three–dimensional nonlinear FE analysis of the internal and external joint types. Jba [24] proposed an improved constitutive model of fiber-reinforced concrete (FRC) diffusion crack and used it to simulate the mechanical behavior of FRC under uniaxial load. The comparison between numerical simulation results and uniaxial loading test results shows that the model can well simulate the mechanical properties of FRC. However, as a new type of beam–column joints, the performance of SFRHC –BCJs is very complex, and the effect of some key parameters (such as concrete strength, steel fiber volume ratio, the stirrup ratio in the core area, etc.) is unknown, which may prevent them from being applied in the engineering practice. Therefore, this paper will attempt to analyze the seismic behavior of BCJs using the ABAQUS program in detail and quantify the influence of potential parameters.

**Figure 1:**
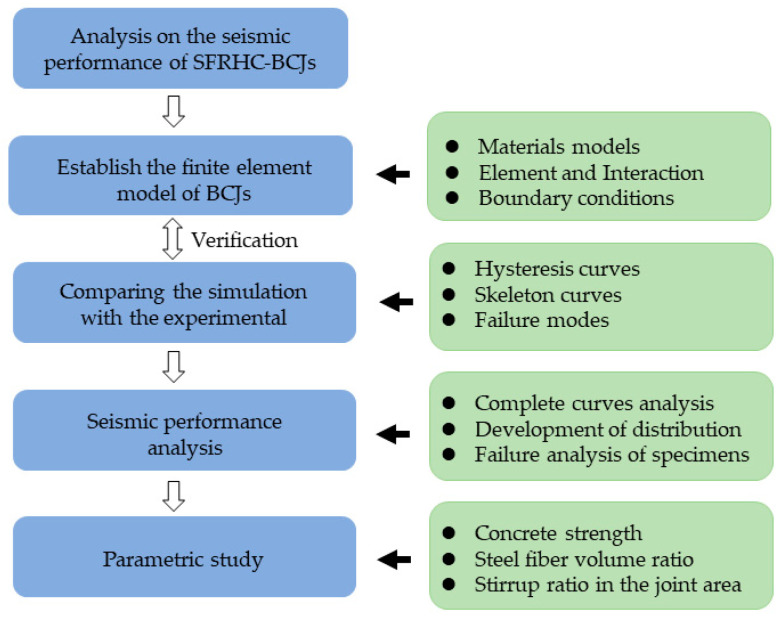
The flowchart of SFRHC–BCJs.

Against this background, this study aims to explore the seismic behavior of BCJs. Based on the experimental and numerical studies in this team, the main contents of this paper are as follows: Firstly, the finite element software ABAQUS was utilized to establish the 3D finite element model of BCJs. Then, the feasibility and accuracy of the numerical simulation were verified by comparing the calculation results with the experimental observation results, and the skeleton curve and failure process were analyzed and clarified in detail according to the simulation results. In addition, based on the stress nephogram, the development of the stress distribution in the concrete and reinforcement during the loading process was clarified comprehensively. Finally, based on the verified FE model, the effect of various parameters on the seismic behavior of BCJs was analyzed in detail and fully evaluated, including concrete strength, steel fiber volume ratio, and stirrup ratio in the core area. Figure 1 shows the flow chart of the study. The research results can reference the optimal design of seismic performance of SFRHC.

## 2. Finite Element (FE) Modeling

In this study, the experimental results and the specifications of the SFRHC as BCJs reported by Shi et al. [25] were used to establish the FE model. The more detailed information for all specimens could be referred to in [25].

### 2.1. Material Model

#### 2.1.1. λ λ Concrete

Thus far, different materials models have been utilized in the numerical modeling of concrete cracking, such as concrete damaged plasticity (CDP), discrete crack model (DCM), and smeared crack model (SCM). In this study, the concrete damage plasticity (CDP) model was adopted to simulate the nonlinear behavior of the concrete elements under multiaxial stress state conditions. That is because the CDP model can capture the degradation of the elastic stiffness in the strain-softening branch of the stress–strain curve under compression (*d_c_*) and tension (*d_t_*). The elastic behavior of concrete material was defined using the elastic isotropy option. The steel fiber-reinforced concrete constitutive model [26,27] was adopted for the concrete in this paper, and the calculation formulas were 1 and 2. The elastic modulus and compressive strength of concrete materials were measured according to the experimental data.

In compression [26],

(1) $$\begin{array}{l} \varepsilon_{cf,r}=(700+172\sqrt{f_{cf}})(1.0+0.189\lambda_{f})\times 10^{-6}\\ \lambda_{f}=V_{f}l_{f}/d_{f}\\ \sigma =(1-d_{cf})E_{cf}\varepsilon \\ d_{cf}=\left\{ \begin{array}{l} 1-\frac{\rho_{cf}n}{n-1+x^{n}}(x\leq 1)\\ 1-\frac{\rho_{cf}}{\alpha_{cf}{\left(x-1\right)}^{2}+x}(x>1) \end{array}\right.\\ \rho_{cf}=\frac{f_{cf,r}}{E_{cf}\varepsilon_{cf,r}}\\ n=\frac{E_{cf}\varepsilon_{cf,r}}{E_{cf}\varepsilon_{cf,r}-f_{cf,r}}\\ x=\varepsilon /\varepsilon_{cf,r}\\ \alpha_{cf}=(0.157f_{cf}{}^{0.785}-0.905)\left[(1-0.0192(l_{f}/d_{f})V_{f}{}^{0.08}\right] \end{array}$$

where *f_cf_* is the compressive strength of SFRHC; *l_f_*, *d_f_*, *V_f_* are the length, diameter, and volume ratio of steel fiber, respectively; *α_cf_* is the shape parameter of the descending section of the stress–strain curve of SFRHC under uniaxial compression; *f_cf,r_* is the compressive strength value of SFRHC; *ε_cf,r_* is the peak compressive strain of SFRHC corresponding to uniaxial compressive strength *f_cf,r_*; *d_cf_* is the damage evolution parameter of SFRHC under uniaxial compression.

In tension [27],

(2) $$\begin{array}{l} f_{tfk}=f_{cfk}/(10.13+0.05\times f_{cfk}-0.5\lambda_{f})\\ \lambda_{f}=V_{f}l_{f}/d_{f}\\ \varepsilon_{tf,r}=f_{tf,r}{}^{0.54}\times 65\times (1+0.2\lambda_{f})\times 10^{-6}\\ \sigma =(1-d_{tf})E_{cf}\varepsilon \\ d_{tf}=\left\{ \begin{array}{l} 1-\rho_{tf}\left[1.2-0.2x^{5}\right],x\leq 1\\ 1-\frac{\rho_{tf}}{\alpha_{tf}{\left(x-1\right)}^{1.7}+x},x>1 \end{array}\right.\\ \rho_{tf}=\frac{f_{tf,r}}{E_{cf}\varepsilon_{tf,r}}\\ x=\frac{\varepsilon }{\varepsilon_{tf,r}}\\ \alpha_{tf}=0.312f_{tf,r}{}^{2}/(1+36\lambda_{f}) \end{array}$$

where *f_cfk_* is the standard value of axial compressive strength of SFRHC; *f_tf,r_* and *ε_tf,r_* are the axial tensile peak stress and corresponding peak strain of SFRHC; *α_tf_* is the shape parameter of the descending section of the stress–strain curve of SFRHC under uniaxial tension; *f_tf,r_* is the representative value of tensile strength of SFRHC; *ε_tf,r_* is the peak tensile strain of SFRHC corresponding to uniaxial tensile strength *f_tf,r_*; *d_tf_* is the damage evolution parameter of SFRHC concrete under uniaxial tension; *E_cf_* is the elastic modulus of SFRHC.

**Figure 2:**
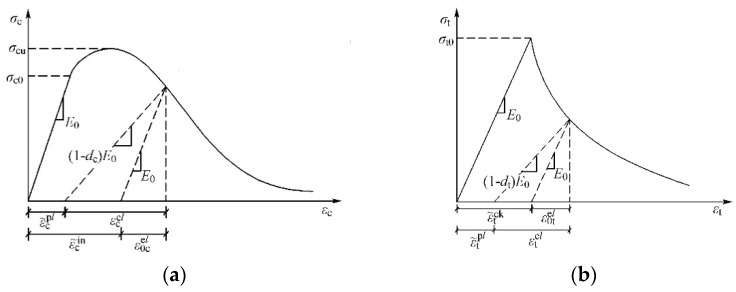
The typical stress–strain relation of the concrete: (**a**) in tension; (**b**) in compression.

Figure 2 shows typical tensile and compressive strain–stress relationships of the concrete material. It shows that the elastic stiffness of the concrete material degraded in the softening section of stress–strain curves. The degradation of the concrete elastic stiffness is simulated using damage parameters in compression (*d_c_*)and tension (*d_t_*) available in the concrete damage plasticity option. Since cyclic loading was applied to the FE model, the degradation of the concrete elastic stiffness was considered for the unloading process. The damage parameters in compression (*d_c_*) and tension (*d_t_*) were calculated using Equations (3) and (4) [28]. Furthermore, the default values of the stiffness recovery coefficient in compression (*w_c_*) and tension (*w_t_*) were considered in the concrete damage.

(3) $$d_{c}=1-\frac{\sigma_{c}}{E(\varepsilon_{c}-\varepsilon_{p})}$$

(4) $$d_{t}=1-\frac{\varepsilon_{cr}}{\varepsilon_{t}}$$

where $\varepsilon_{p}$ is the concrete plastic strain in compression.

The relevant parameters in ABAQUS were defined as follows: the Poisson’s ratio (*v*_c_) is set to 0.2; the eccentricity is 0.1; the ratio of initial equal biaxial compressive yield stress to initial uniaxial compressive yield stress (*f*_b0_/*f*_c0_) is 1.16; the ratio of the second stress invariant on the tensile meridian to that on the compressive meridian is 2/3; the viscosity parameter is taken as 0.005; the dilation angle is 30°. Meanwhile, the default values of the recovery factors of compressive and tensile stiffness *w*_c_ = 0.8 and *w_t_* = 0.2 were used in the calculations, respectively. Furthermore, the compressive damage variable and tensile damage variable [28] were adopted to represent the damage of concrete under cyclic loadings.

#### 2.1.2. λ λ Steel

According to a great number of experimental studies on the material properties of steel, an elastoplastic mixed hardening constitutive model, with consideration of Von Mises yield criteria, Prandtl–Reuss flow rule, and isotropic strain hardening, was adopted to describe the constitutive behavior of the stirrups and longitudinal reinforcement in the FE models. This model was also accounted for the well-known Bauschinger effect [29] for steel under cyclic loading, which was characterized by reduced yield stress upon load reversal after plastic deformation. The yield stress, ultimate stress, and the elongation of the reinforced materials were obtained from the experimental test [25].

### 2.2. FE Models and Boundary Conditions

The commercial software ABAQUS/Standard (v.6.14), which is extensively adopted to analyze the RC structures, was used to establish FE models. In those models, the 8–node reduced integral format 3D solid element (C3D8R), and the 2–node linear 3D truss elements (T3D2) were employed to model the concrete and steel reinforcement, respectively [30,31]. In addition, the embed function was applied for the reinforcement and concrete, where the reinforcement was embedded into the concrete, and no bond–slip relation was defined between the concrete and reinforcements [31,32,33].

**Figure 3:**
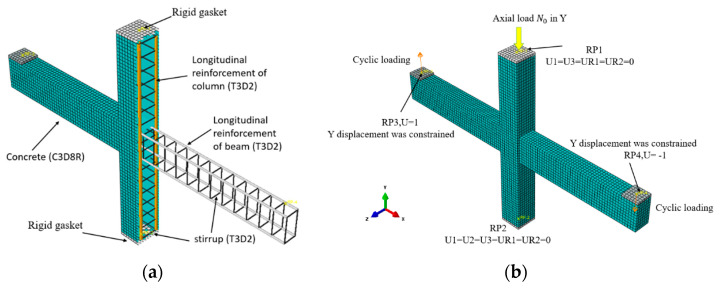
(**a**) Unit type; (**b**) boundary condition and loading of the FE model.

Rigid gaskets were provided at the top and bottom of the column to prevent stress concentration at the loading point. A tie constraint may couple two separate surfaces so that no relative motion would occur. Therefore, the tie option is adopted for the constraint between the rigid gasket and the column end as well as the bottom. All the rigid gaskets were modeled as rigid bodies. The structured meshing technique was adopted, as shown in Figure 3.

The same boundary conditions and loading modes were adopted in the FE models as the pseudo-static tests. Firstly, the top end of the upper column was constrained in X and Z directions at reference point 1, and the axial force was in the Y direction to enable the free movement of the component. Then, the bottom end of the lower column was constrained in X, Y, and Z directions at reference point 2. This simulates the hinge support system at the top and bottom ends of the tested columns. Finally, the free ends of the beam were constrained in the Y direction for imposing the displacement at reference points 3 and 4. The FE modeling is shown in Figure 3.

**Figure 4:**
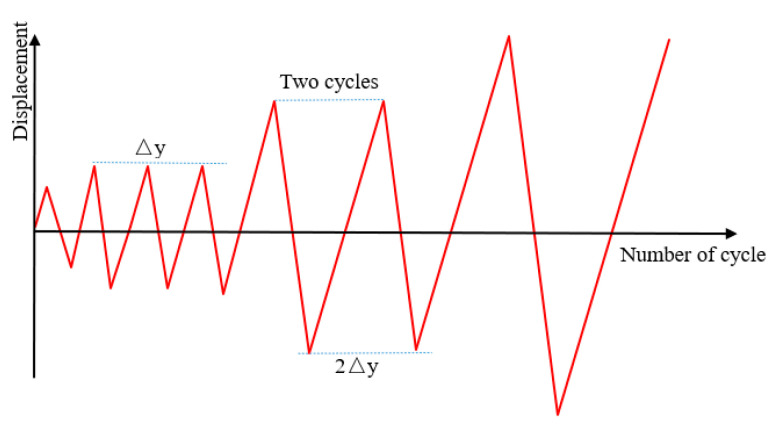
Loading details.

Two loading steps were set in the FE pseudo-static analysis. In the first step, the initial constant axial load *N_0_* was applied to the top of the column in the form of pressure and kept until the end of loading. In the second step, the corresponding cyclic loading was applied to the beam end. In addition, the cyclic displacement loading system was the same as the test. Figure 3 shows the boundary condition and loading of the test specimen. Figure 4 gives the loading details.

### 2.3. Verifications of the FE Modeling

According to the material constitutive models, interaction, and boundary condition as mentioned above, the accuracy of numerical analysis results from established FE models are standardized against the corresponding experimental results in this section in terms of the load vs. displacement hysteresis curves, skeleton curves, ultimate bearing capacity, and failure modes.

#### 2.3.1. λ λ Load vs. Displacement Hysteresis Curves and Peak Load

**Figure 5:**
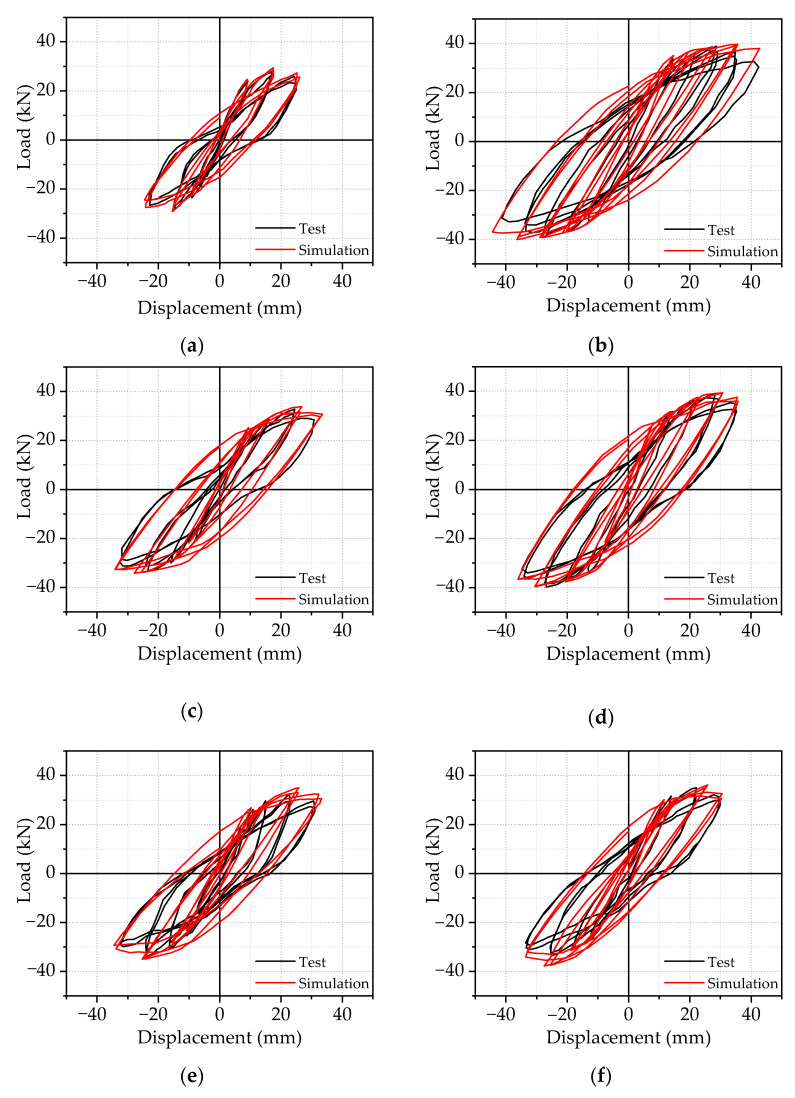

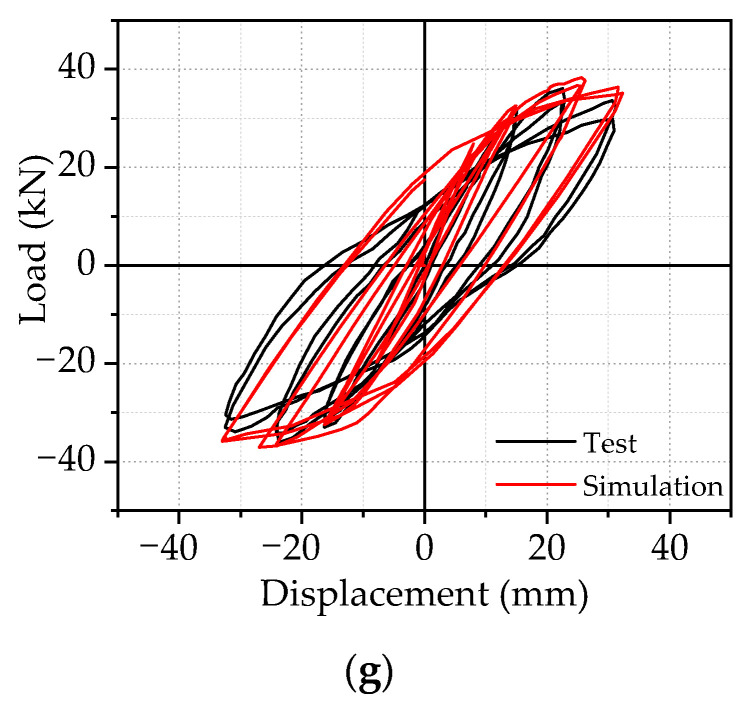
Comparison of hysteresis curves of (**a**) BCJ0; (**b**) BCJ1; (**c**) BCJ2; (**d**) BCJ3; (**e**) BCJ4; (**f**) BCJ5; (**g**) BCJ6.

Figure 5 gives the comparisons of the load vs. displacement hysteretic curves between FE and experimental results. As shown in Figure 5, it can be shown that the overall change trend of theoretical and experimental hysteresis curves of each sample is the same, and the hysteresis curves are relatively complete. Additionally, the hysteresis loops from the numerical analysis are slightly fuller than those from experimental results. That is because FE models were calculated in a perfectly ideal case, where there was no initial defect from the sample prefabrication, meaning that the predicted curves agree reasonably well with the experimental results.

**Figure 6:**
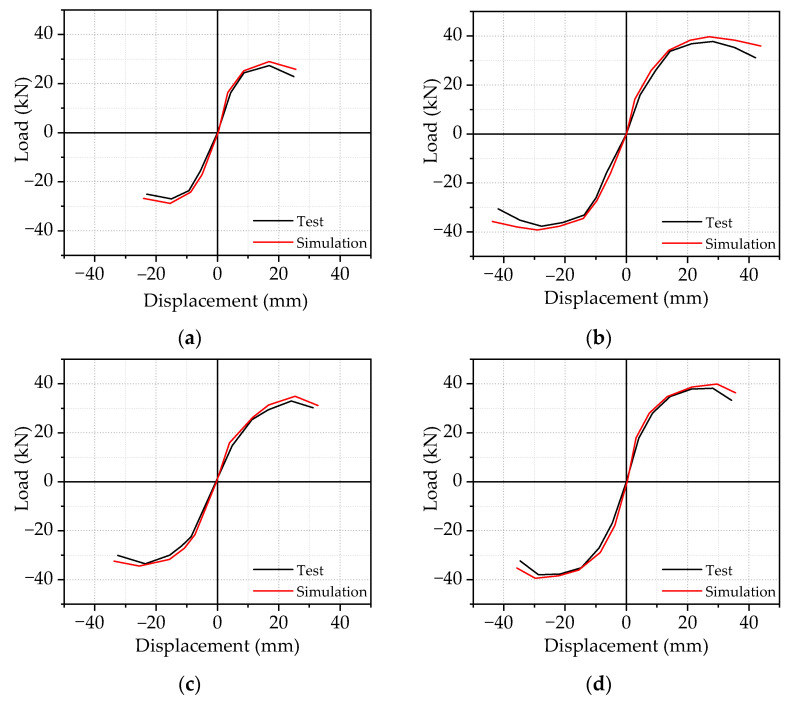

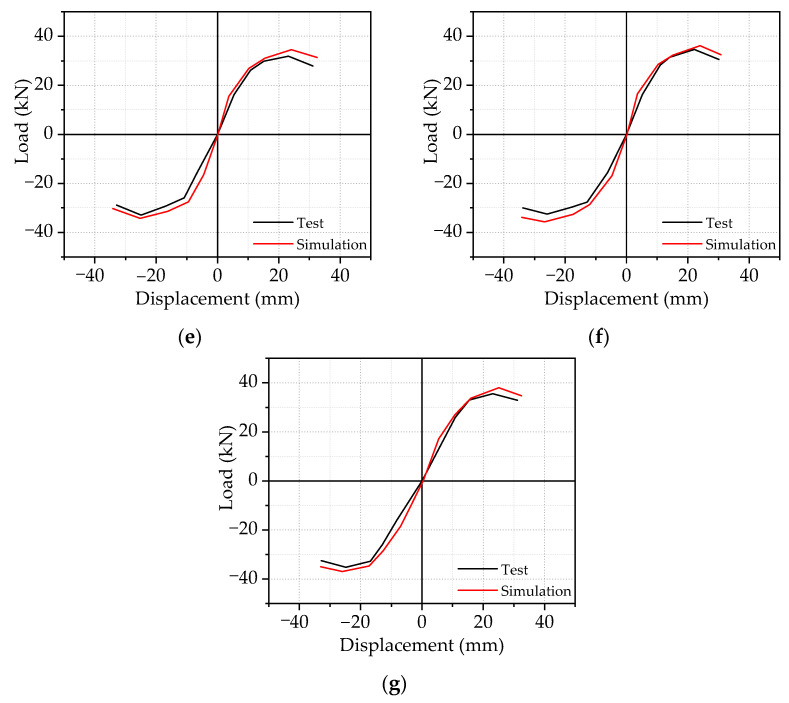
Comparison of skeleton curves of (**a**) BCJ0; (**b**) BCJ1; (**c**) BCJ2; (**d**) BCJ3; (**e**) BCJ4; (**f**) BCJ5; (**g**) BCJ6.

Figure 6 shows the comparison of skeleton curves between the FE and experimental results. The comparisons clearly showed that the skeleton curve of SFRHC specimens could be divided into three stages: elastic stage, elastic–plastic stage, and failure stage. At the initial stage of loading, the specimens were nearly in the elastic stage. The skeleton curve showed a linear growth relationship with a steep slope, indicating that the initial stiffness of specimens was relatively large. With the increase of the applied beam end dis–placement, the slope of the specimens’ skeleton curve decreased, indicating that the specimens’ stiffness decreased continuously and entered the elastoplastic stage. After the peak load, the skeleton curve of the specimen showed a gentle downward trend, which means that the specimens still had good ductility in the later period.

Table 1 summarizes the main features of all simulations developed from FE and failure mode. The main characteristics included yield load *P_y_*, peak load *P_m_*, failure load *P_u_*, the relative errors (RE), and coefficient of variation (COV). The load values for all the specimens are listed in Table 1, which are absolute values of average load values under positive and negative loads. The changing trend of skeleton curves of each specimen reflected the stress situation of each stage, and the skeleton curves achieved good symmetrical performance under both positive and negative loads. Table 1 shows that the finite element calculation results are very close to the test results, and the RE of yield load is 1.3%, and the COV is 0.028. The REs of peak load and failure load are 6.6% and 8.6%, and the COVs are 0.018 and 0.029, respectively. To sum up, the skeleton curve calculated by the finite element method is in good agreement with the experimental results, which further verifies the reliability of the finite element method.

In summary, the FE modeling approach and the material constitutive model adopted in this study are proved to be reasonable and adequate and can be used to investigate the seismic behavior of BCJs further.

#### 2.3.2. λ λ Failure Modes

In this paper, there were two main failure modes of SFRHC: beam-end bending failure (hereafter called BFF) and joint shear failure (hereafter called JSF) in the core area. BFF mainly occurred in specimens with large stirrup ratios and volume ratios of steel fiber. This type of failure was characterized by the crushing of concrete at the compression zone of the beam after yielding tension beam bars, for instance, BCJ1 and BCJ3. JSF mainly occurred in specimens with small stirrup ratios and volume ratios of steel fiber, such as BCJ0, BCJ2, BCJ4, BCJ5, and BCJ6. This type of failure happened due to insufficient strength of stirrups inside the joint to resist the combined shear and tensile forces.

**Figure 7:**
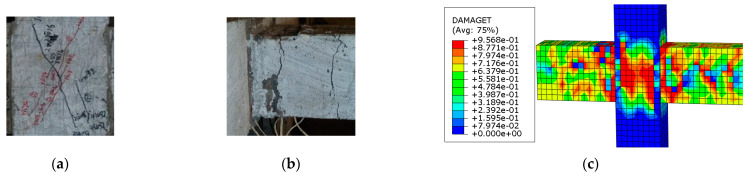
Failure model of BCJ1 specimen: (**a**) in the core joint of test; (**b**) in the beam of test; (**c**) in the FE analysis.

**Figure 8:**
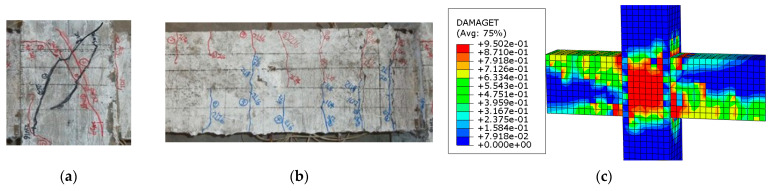
Failure model of BCJ5 specimen: (**a**) in the beam of test; (**b**) in the core joint of test; (**c**) in the FE analysis.

Figure 7 and Figure 8 displays the computed damage distribution versus the observed failure modes of the specimens. The specimen was damaged at the beam end, and a wide crack appeared at the beam end, accompanied by concrete spalling. Fine cracks also appeared in the core area (Figure 7). Figure 8 shows that the core area of the joint was damaged, with wide cracks in the core area, and the concrete in the core area bulged and deformed. There are also tiny cracks at the beam end. It can also be observed that the SFRHC failure mode obtained using numerical analysis is consistent with the test results on the whole. Thus, the FE model fairly represented the cyclic behavior and failure type of beam–column joints.

## 3. Seismic Behavior Analysis and Discussion

Based on the validated FE modeling approach, the FE models are developed to further investigate the seismic performance of beam–column joints in terms of the predicted load vs. displacement hysteric curves during the loading process and failure modes.

### 3.1. Complete Curves Analysis

**Figure 9:**
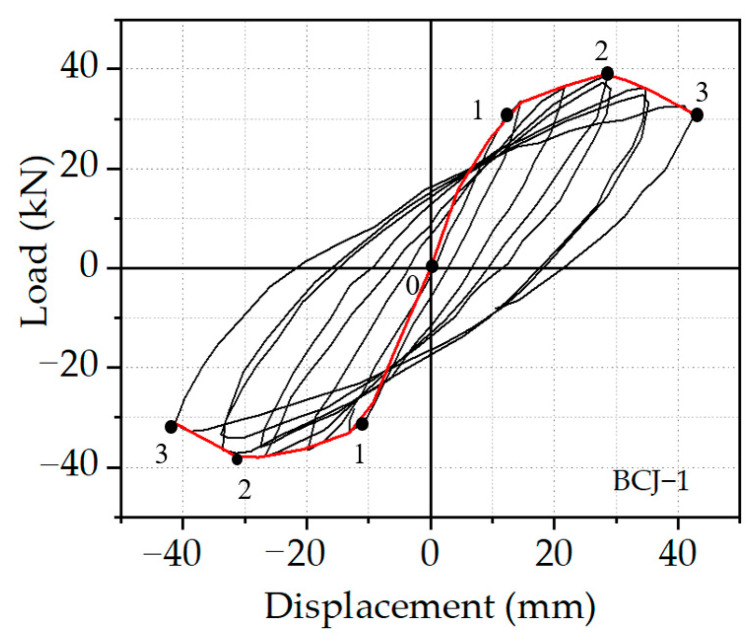
Hysteretic curves and feature points of specimen BCJ1.

Figure 9 shows the hysteric loop from the beam end of the BCJ1 specimen, and three feature points (marked as 1–3 in Figure 9) were chosen from the skeleton curve under both pushing and pulling actions to investigate the load-carrying capacity and deformation capability of each specimen. As shown in Figure 9, It can be seen that the specimen has undergone three stages from loading to failure: elastic stage, elastic–plastic stage, and failure stage. Point 1 stated the yield load point determined by the energy equivalence [34]. Point 2 corresponded to the peak load point, and point 3 was located at the failure load point, corresponding to 85% of the peak load value.

Elastic stage (0–1): At the initial stage of loading, all the specimens were in the elastic phase, indicating that the applied load increased linearly with the increase of the displacement. In this stage, the load increased faster than other stages, whereas the increment of the displacement was very limited. In addition, no obvious deformation of all the specimens was observed, because the vertical load was very small, and the area surrounded by the hysteretic curve was very narrow. The slope of the hysteretic curve changed little, and the residual deformation after unloading was also minimal, indicating that the specimen was in the elastic working state.

Elastic–plastic stage (1–2): When the load reached point 1, many vertical cracks appeared in the concrete at the beam end, which led to the degradation of the stiffness of beam–column joints. With the increase of cyclic load, the concrete in the core area of the joint began to crack, and more cracks appeared at the root of the beam end. The displacement no longer increased linearly with the load, and the specimen entered the elastoplastic stage. The slope of the skeleton curve started to decrease and deviate from the displacement axis. Meanwhile, the stiffness degradation phenomenon was obvious. The concrete strain gradually increased and reached the yield state, and the strain of reinforcement increased accordingly. At this time, the area enclosed by hysteretic curves gradually increased and became fuller, indicating that the energy dissipation capacity of the specimen was increasing.

Failure stage (2–3): After reaching the peak load (point 2), the curve entered the descending stage, and the bearing capacity of the specimen began to decline. The crack at the beam end developed rapidly, while the crack in the core area developed slowly. The strength and stiffness of SFRHC deteriorated seriously until it lost its bearing capacity and was destroyed (point 3). The load–displacement hysteretic curve of the whole beam–column joint was full without the pinch phenomenon, revealing that the steel bar and concrete can work in harmony and deliver good seismic performance.

### 3.2. Failure Analysis of Specimen BCJ1

**Figure 10:**
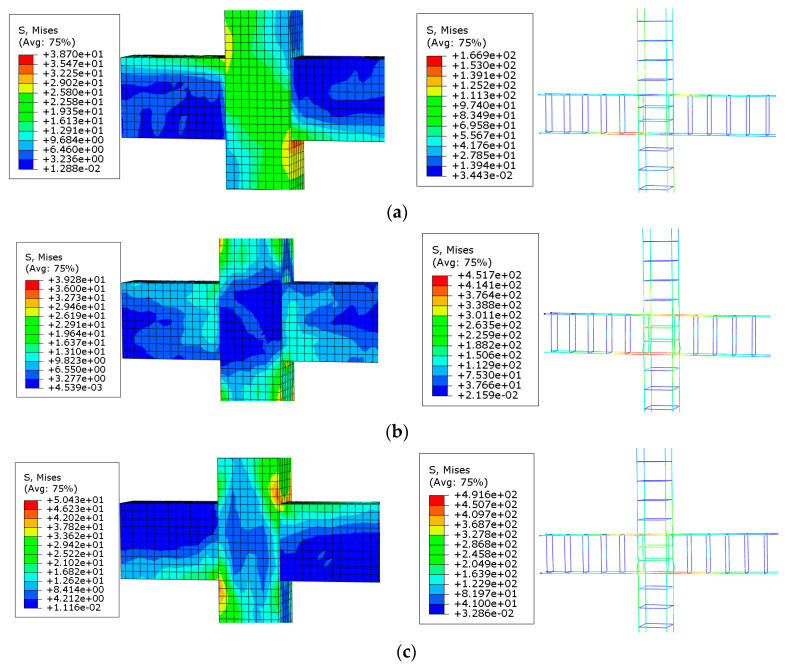
Stress distribution of reinforcement and concrete skeleton of BCJ1: (**a**) yield load; (**b**) peak load; (**c**) failure load.

Figure 10 gives the concrete stress nephogram and reinforcement stress nephogram of BCJ1 specimen. As shown in Figure 10, at the initial loading stage (point 1), the concrete stress at the junction between the compressed beam and the column was large, and the concrete beam end first appeared with fine cracks. With the increase of load, the compressive zone expanded to the end, and the maximum compressive stress of concrete still did not reach the ultimate strength of concrete. The main tensile stress in the joint core area exceeded the tensile strength of concrete, and cracks appeared in the core area of concrete, which was consistent with the phenomenon of inclined cracks in the core area during the test. When the load control stage was loaded to the maximum value (point 2), the stress of the longitudinal reinforcement at the root of the beam yielded, and the core area yielded. Then, numerous cracks appeared in the beam end and core area. When the specimen reached the limit stage (point 3), the stress of the longitudinal reinforcement at the root of the beam yielded, the stirrup at the core area and the stirrup at the end of the beam reached the yield stress, and the specimen was destroyed.

## 4. Parametric Study

Based on the validated FE modeling approach, 12 numerical models were established to extensively investigate the effect of some key parameters on the seismic performance of beam–column joints, including the concrete strength, steel fiber volume ratio, and stirrup in the joint area. The above analysis can be expected to provide a reliable reference for design and application in RC structures.

### 4.1. Effect of Concrete Strength

**Figure 11:**
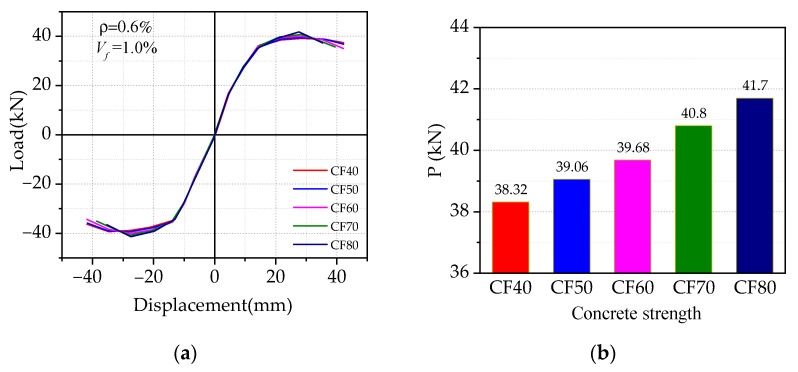
Effect of concrete strengths: (**a**) comparison of skeleton curve; (**b**) comparison of peak load.

Figure 11 gives the effect of concrete strength on the load vs. displacement hysteresis curves, where the concrete strength rings from CF40 to CF80, while others are kept the same. It can be seen from Figure 11 that with the increase of concrete strength, no significant difference of initial stiffness in the skeleton curves was observed in the initial stage, while the peak load was improved by less than 9%, which indicated that increasing concrete strength cannot effectively improve the peak load of SFRHC specimens. However, with the increase of concrete strength, the ductility of the specimen first increased and then decreased gradually. When the concrete strength reaches CF80, the ductility decreased by 13%, which reflected the brittle behavior of high-strength concrete, that is, increasing the concrete strength is not conducive to the seismic ductility of BCJs.

### 4.2. Effect of the Steel Fiber Volume Ratio

**Figure 12:**
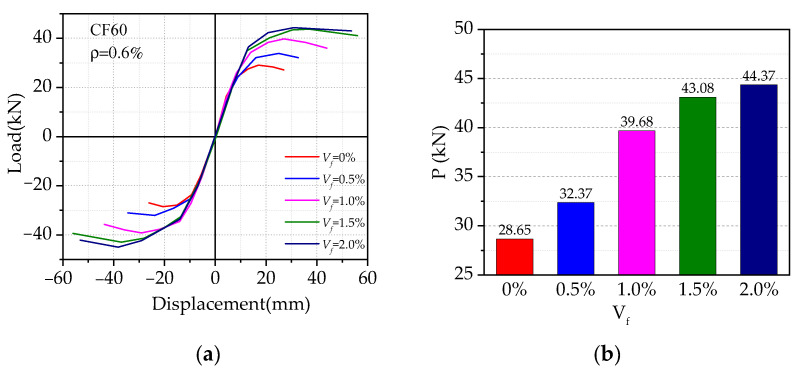
Effect of steel fiber volume ratios: (**a**) comparison of skeleton curve; (**b**) comparison of peak load.

Figure 12 presents the effect of different volume ratios of steel fiber on the load vs. displacement hysteresis curves. The volume ratios of steel fiber (*V_f_*) ranged from 0 to 2%, while others were kept the same. As shown in Figure 12, it can be found that steel fiber can significantly improve the seismic performance of SFRHC beam–column joints, in terms of peak load and ductility. With the increase of steel fiber volume ratio, the initial stiffness of the joint was increased by 2%, while the unloading stiffness of each joint was relatively small. With the increase of steel fiber volume ratio, the slope of each model skeleton curve changed in the elastic section and the falling section, and the overall impact was minor. In comparison to the specimen without steel fiber, the peak load of specimens with 0.5%, 1.0%, 1.5%, and 2.0% steel fiber volume ratios were improved by 13%, 38%, 50%, and 55%, respectively, and the corresponding ductility were improved by 18%, 61%, 102%, and 91%. This indicates that before the volume ratio of steel fiber increases to 1.5%, the seismic performance increases rapidly. When the volume ratio of steel fiber exceeds 1.5%, the ductility decreases.

### 4.3. Effect of Stirrup Ratio in the Joint Area

**Figure 13:**
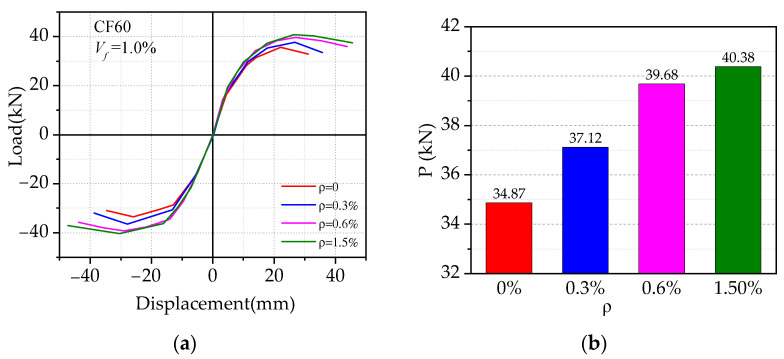
Effect of the stirrup ratios in the core area: (**a**) comparison of skeleton curve; (**b**) comparison of peak load.

Figure 13 shows the effect of varied stirrup ratios on the load vs. displacement hysteresis curves, where the stirrup ratio ranged from 0 to 1.5%, while others were kept the same. As shown in Figure 13, it can be found that the initial tangential stiffness of BCJs with different stirrup ratios almost remained unchanged, while the larger stirrup ratio delayed the failure and improved the deformation ability. The stirrup ratio can significantly improve the peak load and ductility of SFRHC beam–column joints. When the stirrup ratio increased from 0% to 0.6%, the peak load and ductility of the specimen increased by 14% and 30%, respectively. Meanwhile, it shows a negligible effect on the skeleton curves until the stirrup ratio enlarges to a certain level such as 0.6% in the current study. This may imply that steel fiber can effectively reduce the use of transverse reinforcement, thus reducing the complexity of construction.

## 5. Conclusions

Based on the validated FE modeling approach, this paper aimed to present a numerical investigation on the seismic behavior of SFRHC–BCJs in terms of complete curves analysis, failure mode, parametric study, etc. The main conclusions can be drawn based on the limited research work, as follows:

- The reasonable agreement between experimental and corresponding FE results is achieved generally, and therefore, the FE modeling approach can be adopted to further investigate the seismic behavior of SFRHC–BCJs.
- Based on the predicted load vs. displacement hysteresis curves, the specimens are considered to experience three stages during the loading process: elastic stage, elastic–plastic stage, and failure stage. In addition, the failure modes and processes were analyzed and clarified in detail, including the beam-end bending failure (BFF) and joint shear failure (JSF) in the core area.
- Based on the stress nephogram, the development of the stress distribution in the concrete and reinforcement during the loading process was clarified comprehensively.
- Increasing the concrete strength can slightly improve the ultimate load of BCJs, while the ductility decreased noticeably. Additionally, adding the steel fiber into concrete can clearly improve the seismic performance of the structure. Moreover, increasing the stirrup ratio in the core area can effectively improve the seismic performance of BCJs, and change the failure mode of BCJs.

## Figures and Tables

**Table 1:** Comparisons between simulated and experimental values.

Component Number	*P_y_*/kN			*P_m_*/kN			*P_u_*/kN		
	S	T	S/T	S	T	S/T	S	T	S/T
BCJ0	24.32	23.34	1.042	29.13	27.35	1.065	24.76	22.02	1.124
BCJ1	27.34	28.36	0.964	39.68	37.19	1.067	35.10	31.22	1.124
BCJ2	27.32	26.73	1.022	34.68	33.25	1.043	30.20	27.43	1.101
BCJ3	29.68	29.81	0.996	39.72	37.91	1.048	33.76	32.39	1.042
BCJ4	27.94	26.49	1.055	34.33	32.51	1.056	29.18	27.68	1.054
BCJ5	27.02	26.87	1.006	36.97	33.58	1.101	31.42	29.53	1.064
BCJ6	28.15	27.92	1.008	38.28	35.42	1.081	32.54	29.8	1.092
RE			0.013			0.066			0.086
COV			0.028			0.018			0.029

Note: S represents simulation, T represents test, RE represents the relative errors, COV represents coefficient of variation.

## Data Availability

Data are available on request from the authors.

## Nomenclature

SFRHC	Steel fiber-reinforced high-strength concrete
BCJs	Beam–column joints
FE	Finite element
BFF	Beam-end bending failure
JSF	Joint shear failure
FRC	Fiber-reinforced concrete
CDP	Concrete damaged plasticity
DCM	Discrete crack model
SCM	Smeared crack model
